# The combination of berberine and evodiamine ameliorates high-fat diet-induced non-alcoholic fatty liver disease associated with modulation of gut microbiota in rats

**DOI:** 10.1590/1414-431X2022e12096

**Published:** 2022-05-16

**Authors:** Yufan Dai, Wenyu Zhu, Jiaxuan Zhou, Tao Shen

**Affiliations:** 1 College of Basic Medicine, Chengdu University of Traditional Chinese Medicine, Chengdu, China; 2 Siemens PLM Software, Chengdu, China

**Keywords:** Nonalcoholic fatty liver disease, Gut microbiota, Intestinal barrier, Inflammation, Berberine, Evodiamine

## Abstract

Nonalcoholic fatty liver disease (NAFLD) is considered to be a manifestation of hepatic metabolic syndrome. Some studies on the pathogenesis of NAFLD by targeting gut microbiota have attracted wide attention. Previous studies have demonstrated the positive effects of berberine and evodiamine on metabolic diseases and gut microbiota dysbiosis. However, it is not known whether the combination of berberine and evodiamine (BE) can prevent the development of high-fat diet (HFD)-induced NAFLD. Therefore, we aimed to explore the protective effects of BE on the development of HFD-induced NAFLD from the perspective of the gut microbiota. Gut microbiota profiles were established by high throughput sequencing of the bacterial 16S ribosomal RNA gene. The effects of BE on liver and intestinal tissue, intestinal barrier integrity, and hepatic inflammation were also investigated. The results showed that the abundance and diversity of gut microbiota were enriched by BE treatment, with an increase in beneficial bacteria, such as Lactobacillus, Ruminococcus, and Prevotella, and a decrease in pathogenic bacteria such as Fusobacterium and Lachnospira. In addition, BE effectively improved liver fat accumulation and tissue damage, inhibited the apoptosis of intestinal epithelial cells, increased the contents of intestinal tight junction proteins, and decreased the expression of pro-inflammatory factors. Consequently, BE treatment could be an effective and alternative strategy for alleviating NAFLD by modulating gut microbiota and safeguarding the intestinal barrier.

## Introduction

Nonalcoholic fatty liver disease (NAFLD) is a clinicopathological syndrome characterized by diffuse hepatocyte bullous steatosis that is not caused by alcohol and other factors that cause liver damage. NAFLD comprises a spectrum of liver diseases from simple steatosis to non-alcoholic steatohepatitis, fibrosis, cirrhosis, and eventually hepatocellular carcinoma. The global incidence of NAFLD is about 25% and the prevalence is on the rise (1). Yet, there is still no approved drug therapy for NAFLD.

Gut microbiota plays an important role in regulating host physiology and metabolism. When the stability and diversity of the intestinal bacterial community are disrupted, various human diseases such as obesity, type 2 diabetes, and metabolic syndrome occur (2). A growing body of research has revealed that the dysbiosis of gut microbiota is associated with NAFLD (3) and its severity (4). Professor Marshall described the pathophysiological state between liver lesions and intestinal injury as “enterohepatic axis”, and the application of this theory can explain the dysbiosis of the gut microbiota, the damage of the intestinal barrier in the development of NAFLD, the activation of liver innate immune system, and the increase of hepatocyte inflammatory response (5). Some studies have indicated that a high-fat diet (HFD) alters the components of gut microbiota, induces intestinal barrier malfunction, and increases the intestinal permeability and inflammation (6). Thus, HFD-induced NAFLD can be treated by targeting the dysbiosis of gut microbiota caused intestinal barrier malfunction (7).

The herbal medicine pair Coptidis Rhizoma and Evodia Fructus presented in the Danxi's Mastery of Medicine by Zhu Zhenheng in the 14^th^ century has been used to treat liver and gastrointestinal disorders for hundreds of years since the Yuan Dynasty. The herbal medicine pair Coptidis Rhizoma and Evodia Fructus is officially listed in the *Chinese Pharmacopoeia* as a prescription employed in patients suffering from viral hepatitis, cholangitis, and gastric ulcer, among other disorders (8). Berberine and evodiamine are, respectively, the most important alkaloids of Rhizoma Coptidis and Evodia Fructus, which possess various pharmacological effects such as anti-microbial, anti-inflammatory, and anti-obesity (9-11). The efficacy of berberine as a hypolipidemic and NAFLD protectant has been demonstrated in previous animal and human studies, thus making it a potential drug for the cure of NAFLD (12,13). In addition, evodiamine can promote lipolysis and modulate serum free fatty acids (FFAs) level (14). However, it is not clear whether the combination of berberine and evodiamine (BE) can attenuate NAFLD, and its mechanism remains undefined.

In the current study, we aimed to explore the protective effects of BE on the development of HFD-induced NAFLD from the perspective of the gut microbiota.

## Material and Methods

### Reagents

Berberine and evodiamine (purity >99%) were supplied by Kangdele Pharmaceutical Enterprise (China). Fenofibrate was obtained from Affiliated Hospital of Chengdu University of Traditional Chinese Medicine (29681, Recipharm Fontaine, France). Sodium carboxymethyl cellulose was obtained from Sigma-Aldrich Co., Ltd. (USA).

### Animals

Male Sprague Dawley rats weighing 200-220 g (10 weeks old) were purchased from Hunan SJA Laboratory Animal Co., Ltd., China (SCXK 2019-0004). The animals were housed six per cage with free access to food and drinking water in an environmentally controlled room at 24±2°C, with 55±5% relative humidity and a 12-h light/dark cycle. All rats were fed with a standard basic diet as familiarization for 1 week before the start of the experiment. All animal experiments met ethical standards, and the protocols strictly adhered to the care and licensing guidelines for laboratory animals established by the Institutional Animal Ethics Committee of Chengdu University of Traditional Chinese Medicine (TCM 2016-312).

### Animal experiments

**Figure 1:**
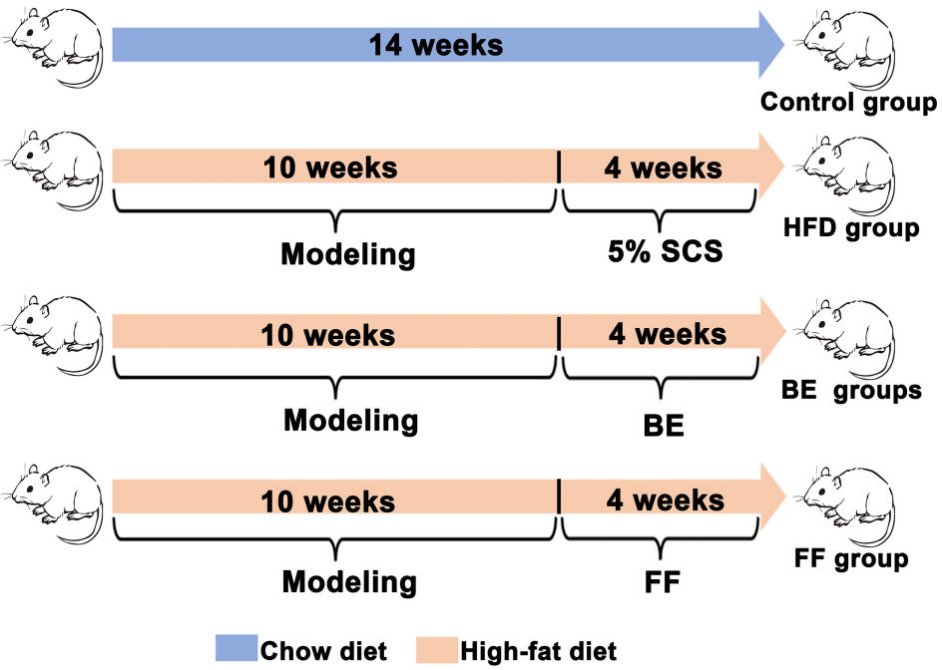
Experimental design. SCS: sodium carboxymethyl cellulose; BE: berberine and evodiamine; FF: fenofibrate.

After an accommodation period of 1 week, a total of 60 rats were randomly separated into two groups: the control group (n=10) was fed normal chow, while the NAFLD model group (n=50) was fed the HFD to induce NAFLD for 10 weeks. The HFD contained a normal diet supplemented with 0.2% propylthiouracil, 1% cholesterol, 1% sodium tauroglycocholate, 5% yolk powder, and 10% lard (Ennsville Biotechnology Co., Ltd., China). After 10 weeks of feeding, the NAFLD model group (n=50) was allocated to five groups by systematic sampling: three groups (n=10 in each group) received a combination (BE) of berberine (BB) and evodiamine (EV) at high dosages (HFD-BEH - 72 mg BB/kg bw, 16 mg EV/kg bw) (11), a medium dosage (HFD-BEM, 36 mg BB/kg bw, 8 mg EV/kg bw), and a low dosage (HFD-BEL, 18 mg BB/kg bw, 4 mg EV/kg bw); a positive control group (n=10) received fenofibrate (HFD-FF, 100 mg/kg bw); and a disease model group (HFD group, n=10) received 0.5% sodium carboxymethyl cellulose. Fenofibrate and BE combinations were separately dissolved in 0.5% sodium carboxymethyl cellulose. The respective drugs were administrated to the five groups simultaneously for 4 weeks with HFD. The experimental design is shown in Figure 1.

All experimental rats were weighed twice a week. Fecal samples were collected at the 14th week, immediately frozen in liquid nitrogen, and stored at -80°C. All animals were fasted for 24 h and then weighed and anesthetized with sodium pentobarbital before sacrifice to minimize experimental damage.

### Biochemical assays

The collected blood was centrifuged at 1788.8 *g* for 10 min at 4°C to obtain the serum. The levels of total cholesterol (TC), triglyceride (TG), alanine aminotransferase (ALT), alkaline phosphatase (ALP), aspartate aminotransferase (AST), and γ-glutamyl transferase (γ-GT) were measured in serum with commercial kits (Nanjing Jiancheng Bioengineering Institute, China) following the manufacturer’s instructions.

### Oil-Red-O staining

Liver and ileum samples were collected, sectioned, and fixed with 10% neutral formaldehyde for 30 min, then incubated with 60% isopropanol solution for 5 min, and stained with Oil-Red-O solution for 10 min. After washing the samples, the cell nuclei were stained with hematoxylin for 2 min. The images were analyzed using the Image-Pro Plus 6.0 software (Media Cybernetics, USA), where the percentage of the Oil-Red-O-positive area was calculated per image area to yield the relative staining level for each sample.

### Histopathological analysis

The liver and ileum samples were fixed in 4% paraformaldehyde, cut, dehydrated, embedded, sliced, and stained by hematoxylin and eosin (HE) (Wuhan Google Biotechnology Co., Ltd., China). The hepatic and intestinal tissue sections were viewed under an orthotopic light microscope (Nikon Eclipse E100, Nikon, Japan).

### 16S rRNA sequencing

The sequencing of 16S rRNA was conducted according to the instructions of Zymo Research BIOMICS DNA Microprep Kit (Catalog #D4301, USA). The purity of the extracted genomic DNA was detected by 0.8% rose gel electrophoresis, and then the concentration was detected by PicoGreen dye method. According to the sequencing region, specific primers with index sequences were synthesized to amplify the 16S rDNA V3-V4 region of the sample. The sequences of amplified primers are as follows: 515F (5′-GTGYCAGCMGCCGCGGTAA-3′) and 806R (5′-GGACTACHVGGGTWTCTAAT-3′). After PCR product detection, purification, and quantification, the library was constructed by NEBNext Ultra II DNA Library Prep Kit for Illumina (NEB#E7645L; New England BioLabs Ltd., UK), and then high-throughput sequencing was conducted using Illumina Miseq PE250 platform. Effective tags were obtained by data quality control with the Quantitative Insights Into Microbial Ecology (QIIME) platform (http://qiime.org). Based on the Usearch software (http://www.drive5.com/usearch), operational taxonomic unit (OTU) clustering was performed with UPARSE algorithm at 97% consistency level, and then the representative sequences of OTU were classified by UCLUST classification method. The data were transformed to obtain the relative abundance table of each sample at each classification taxa, including phylum, class, order, family, genus, and species. Alpha and beta diversity analysis were performed using R language. The important bacterial species among different groups were discriminated using the linear discriminant analysis effect size (LEfSe) algorithm (https://galaxyproject.org).

### Quantitative real time polymerase chain reaction (qRT-PCR) analysis

The mRNA expression of intestine fatty acid binding protein (I-FABP) and the tight junction (occludin and Zonula occludens-1 (ZO-1)) in the ileum were assayed by qRT-PCR (15). The total RNA was extracted from the ileum tissue with the TRIzol reagent (Catalog No. 15596018; Thermo Scientific, USA). The total RNA was reverse-transcribed to cDNA (Catalog No. #G492; Applied Biological Materials Inc., Canada). The quantitative PCR was implemented using EvaGreen Express 2X qPCR MasterMix-No Dye (Catalog No. 0194844830001; Applied Biological Materials Inc.). The DNA samples were pre-denatured at 95°C for 10 min (first stage) and then amplified with 40 cycles at 95°C for 15 s, at 60°C for 60 s, and the final extension from 60°C to 95°C, rising 0.3°C every 15 s. The relative mRNA expression levels were computed with the 2^-ΔΔCt^ method, and the Ct values were normalized using β-actin as a reference gene. The primers used were designed and validated by Sangon Biotech Co., Ltd. (China) and are listed in Table 1.

**Table 1:** Primer sequences used for RT-qPCR assay.

Genes	Forward (5′-3′)	Reverse (5′-3′)
Actin	TGTCACCAACTGGGACGATA	GGGGTGTTGAAGGTCTCAAA
I-FABP	ATGGGCATTAACGTGGTGAAGAGG	GACGCCGAGTTCAAACACAACATC
ZO-1	CGCAGCCAGTTCAAACAAAGTTCC	GCAACATCAGCAATCGGTCCAAAG
Occludin	ATGCACGTTCGACCAATGCT	GGATCCGAATCACCCCTGGA

I-FABP: intestine fatty acid binding protein; ZO-1: zonula occludens-1.

### Ileum epithelial cells apoptosis

Terminal-deoxynucleotidyl Transferase Mediated Nick End Labeling (TUNEL) staining was used to detect the apoptosis of ileum epithelial cells according to previous research (16). Terminal ileum slides were stained and detected in accordance with the manufacturer's instructions for the TUNEL assay kit. Paraffin sections were dewaxed, hydrated by gradient ethanol, permeated by protease K for 20 min, and washed 3 times with PBS for 5 min each, according to the Fluorescein (FITC) Tunel Cell Apoptosis Detection Kit (Servicebio Biotechnology Co., Ltd., China). The samples were incubated at 37°C for 1 h and protected from light, according to the manufacturer's instructions. The positive and negative controls consisting of intestinal samples were treated with 0.1 mg/mL of DNaseor or incubated without TdT, respectively.

### Enzyme linked immunosorbent assay (ELISA)

The liver tissue was ground with a grinder, homogenized under ultrasound (10% w/v), and centrifuged at 1788.8 *g* for 10 min at 4°C. Lipopolysaccharide (LPS), tumor necrosis factor-α (TNF-α), interleukin (IL)-1β, IL-6, IL-10, and IL-4 were assessed by ELISA Kits (MultiScience (Lianke) Biotechnology Co., Ltd., China). The levels of malondialdehyde (MDA) (Nanjing Jiancheng Bioengineering Institute) and superoxide dismutase (SOD) (Elabscience Biotechnology Co., Ltd., China) were detected according to manufacturer instructions. The content of total protein was confirmed by BCA protein analysis kits (MultiScience (Lianke) Biotechnology Co., Ltd.).

### Statistical analysis

All results are reported as means±SD, and analyses were performed using Prism 8.0.2 (GraphPad Software Inc., USA). Differences between two groups were carried with the unpaired two-tailed Student's *t*-test. For more than two groups, one-way analysis of variance (ANOVA) was used, followed by the Bonferroni's *post hoc* test. A bioinformatics analysis was performed including species taxonomy, richness, and diversity analysis. P values <0.05 were considered to be statistically significant.

## Results

### Effects of BE on hepatocyte steatosis and hepatic injury

**Figure 2:**
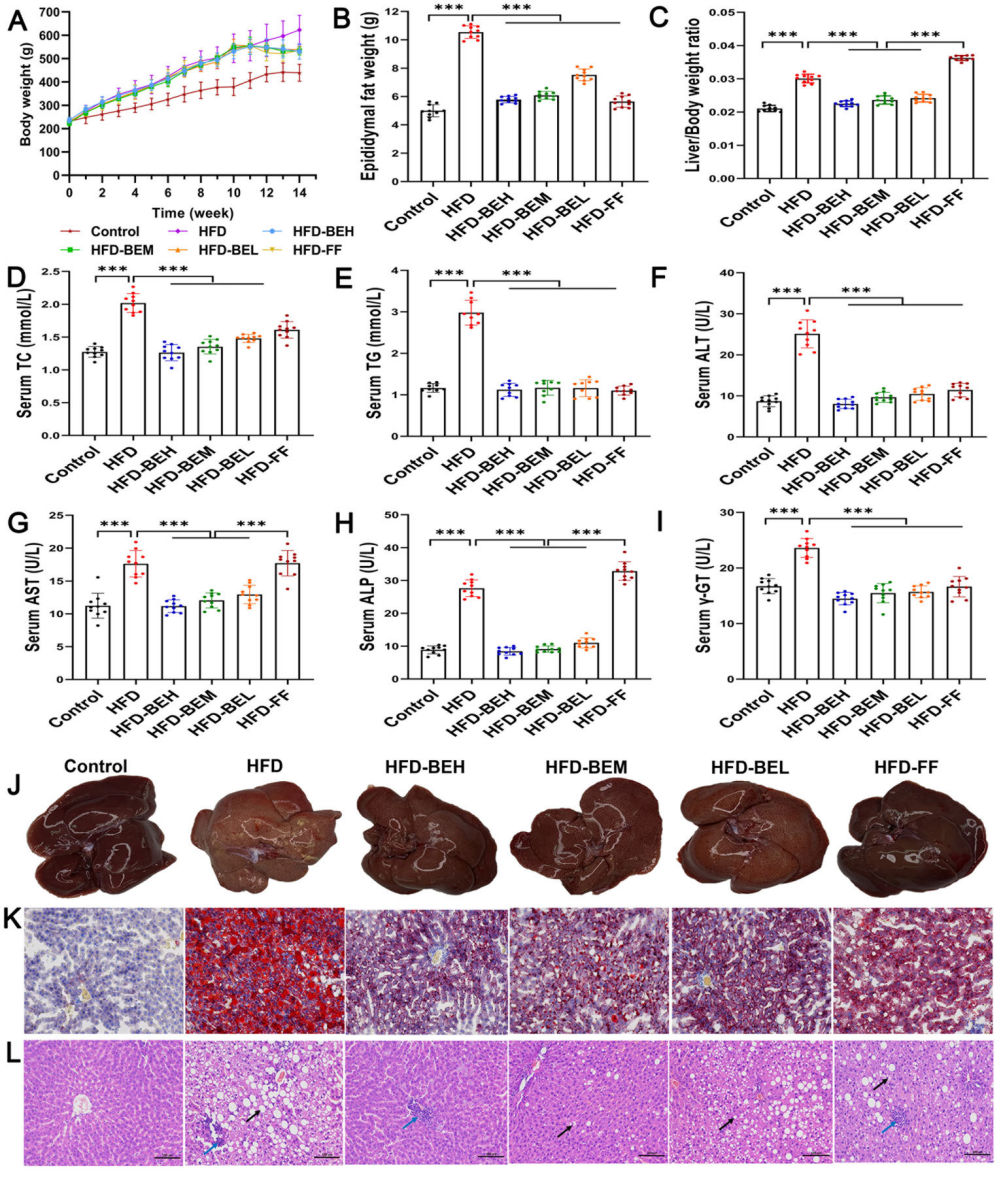
Effect of berberine and evodiamine (BE) at high, medium, and low doses (BEH, BEM, BEL) or fenofibrate (FF) on lipid accumulation and pathological lesions of the liver of animals fed a high-fat diet (HFD). **A**, Changes in body weights, (**B**) epididymal fat weights, and (**C**) liver/body weight ratio. Serum levels of (**D**) triglyceride (TC), (**E**) total cholesterol (TG), (**F**) alanine aminotransferase (ALT), (**G**) aspartate aminotransferase (AST), (**H**) alkaline phosphatase (ALP), and (**I**) γ-glutamyl transferase (γ-GT). **J**, Representative macroscopic images of livers; **K**, Oil-Red-O staining; and **L**, hematoxylin and eosin (HE) staining images of the liver (magnification: 200×, scale bar: 100 µm). The black arrows represent circular vacuoles; the blue arrows represent inflammatory cell infiltration. Data are reported as means±SD. ***P<0.001 (one-way ANOVA).

In comparison with the HFD group, the three BE treatment groups showed an extraordinary decrease in body weight, epididymal fat weight, and liver index (P<0.01) (Figure 2A-C). Moreover, TC and TG contents decreased dramatically, as two sensitive indexes for BE effect on lipid accumulation in the liver (Figure 2D, E). In addition, BE supplementation significantly alleviated liver injury, as confirmed by ALT, AST, ALP, and γ-GT, superior to fenofibrate for AST and ALP (P<0.001) (Figure 2F-I). The representative macroscopic images and Oil-Red-O staining similarly revealed the reduction of liver fat accumulation (Figure 2J, K). The degree of red staining and concentration of lipid droplets in liver tissue were significantly improved. HE staining indicated pathological changes of the liver after BE intervention (Figure 2L). A small number of hepatocytes steatosis and round vacuoles were observed in the cytoplasm, while a small amount of inflammatory cell infiltration was observed in the tissues.

In a nutshell, our current results confirmed that BE can significantly exert a protective role on HFD-induced NAFLD.

### Effects of BE on the diversity of gut microbiota

**Figure 3:**
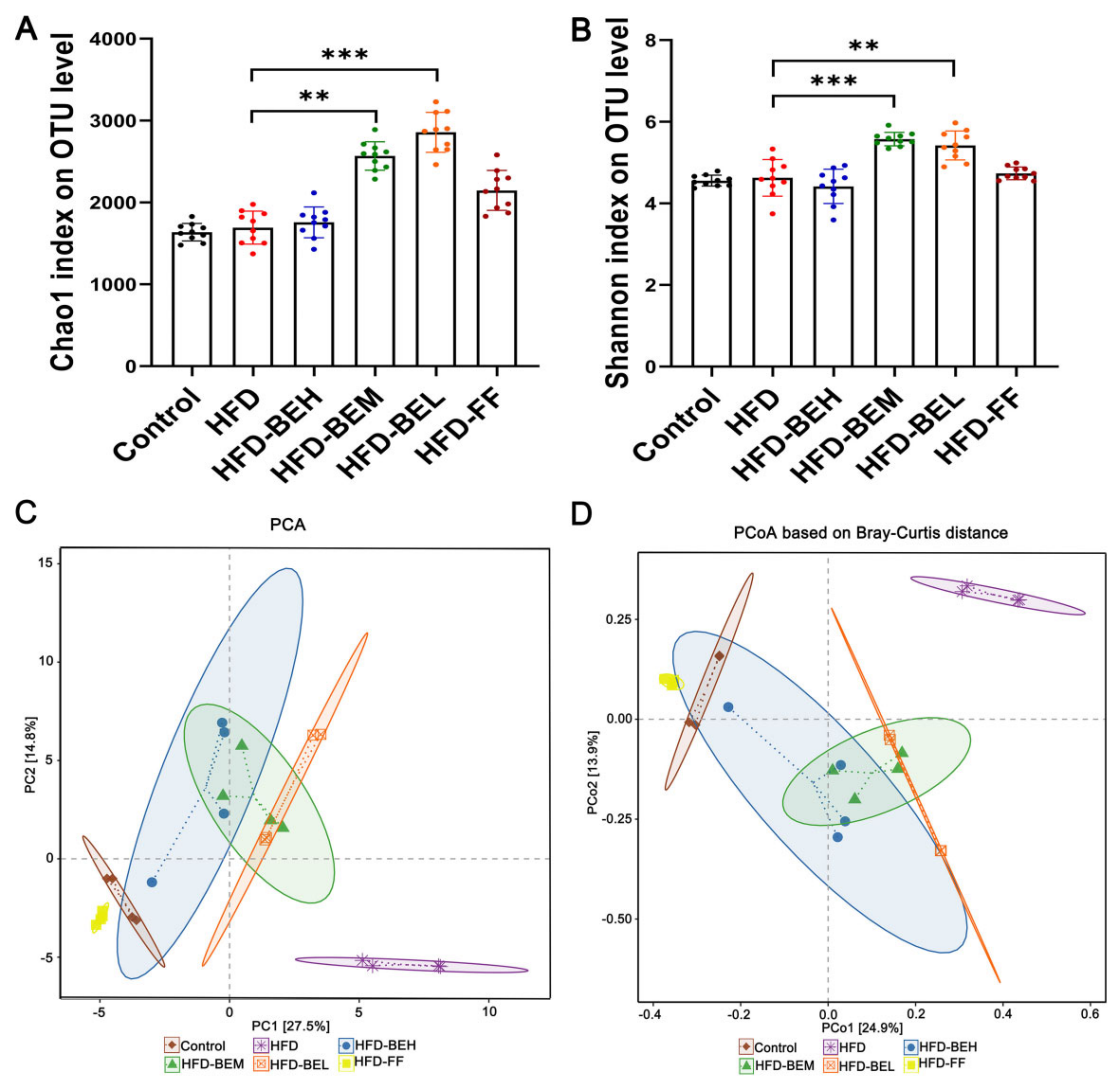
Effect of berberine and evodiamine (BE) at high, medium, and low doses (BEH, BEM, BEL) or fenofibrate (FF) on the diversity of gut microbiota of animals fed a high-fat diet (HFD). Alpha diversity analysis of Chao1 (**A**) and Shannon indexes (**B**) on operational taxonomic units (OTU). **C**, Beta diversity analysis, principal component analysis (PCA) plot and (**D**) principal co-ordinates analysis (PCoA) based on Bray-Curtis distances on OTU level. Data are reported as means±SD. **P<0.01, ***P<0.001 (one-way ANOVA).

To determine whether the therapeutic effects of BE are related to the regulation of gut microbiota, we analyzed the fecal bacteria 16S rRNA gene in HFD-fed rats. The Coverage index of each group greater than 0.9 showed that the sample sequencing depth in this experiment was sufficient and the results were highly reliable and reasonably available. Compared with the HFD group, BE treatment groups (at medium and low dosages) can significantly magnify abundance and diversity of gut microbiota in line with Chao1 (P<0.01 or P<0.001) and Shannon indexes (P<0.001 or P<0.01) (Figure 3A, B). In the HFD group, Chao1 and Shannon indexes were increased to a certain extent, but their differences were not statistically significant (Figure 3A, B). In order to easily observe the difference and variation of among samples, principal component analysis (PCA) was first used for exploration (Figure 3C). Subsequently, principal co-ordinates analysis (PCoA) was used based on the bray-Curtis distance matrix to analyze Beta diversity (Figure 3D). The consequences of PCA and PCoA showed that the clusters of gut microbiota in the BE treatment groups were visibly different from the HFD group, and closer to the control group. Taken together, these findings suggested that BE complement can regulate the Alpha and Beta diversity of gut microbiota in HFD-induced rats.

### Effects of BE on the structure of gut microbiota

**Figure 4:**
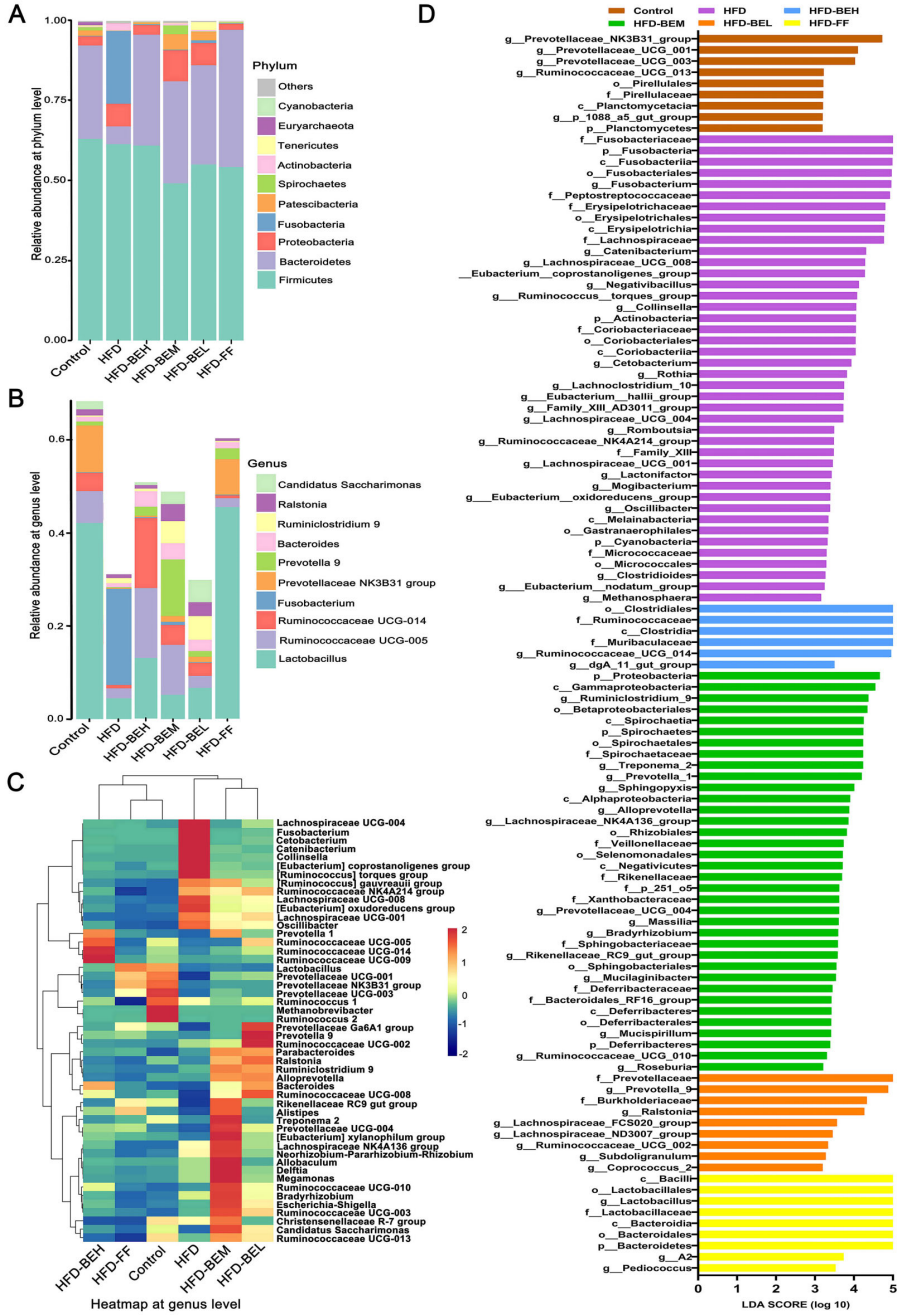
Effect of berberine and evodiamine (BE) at high, medium, and low doses (BEH, BEM, BEL) or fenofibrate (FF) on the structure of gut microbiota animals fed a high-fat diet (HFD). Bacterial taxa of relative abundance at (**A**) phylum level and (**B**) genus level. **C**, Heatmap of abundance at genus level. **D**, Linear discriminant analysis (LDA) effect size (LEfSe) score.

To further reveal the differences in community composition among samples, we analyzed the related abundance of the five most abundant phyla and genera. At the level of phylum, the results demonstrated that gut microbiota was mainly composed of Firmicutes, Bacteroidetes, Proteobacteria, Fusobacteria, and Patescibacteria (Figure 4A). In contrast to the HFD group, BE reversed the trend of HFD in reducing the proportions of Bacteroidetes, and the ratio of Firmicutes to Bacteroidetes (F/B) was also decreased (all P<0.05) (Figure 4A). Notably, HFD consumption vastly increased the proportion of Fusobacteria in the HFD group, and it was not a major component in other groups. In terms of genus level, the control group was mainly composed of Lactobacillus, Prevotellaceae-NK3B31 group, Ruminococcaceae-UCG-005, Ruminococcaceae-UCG-014, and Candidatus-Saccharimonas, whose proportions were limited in the HFD group (Figure 4B). Whereas the contents of Fusobacterium were highly expressed in the HFD group. However, BE intervention increased the relative quantity of Lactobacillus, Prevotella-9, Ruminococcaceae-UCG-005, Ruminococcaceae-UCG-014, and Bacteroides. Then, based on the abundance of OTUs, the top 50 bacteria were classified and clustered at the genus level, and the gut microbiota among groups were analyzed by comparing the heat map (Figure 4C). We detected that the tendency of these bacteria, including Lactobacillus, Ruminococcus, Prevotella, Fusobacterium, and Bacteroides, were in accordance with the previous results. Noticeably, Lachnospira was also highly expressed in the HFD group. The above results showed that BE complement altered the community compositions of gut microbiota, which was comparable to the control group.

LEfSe was used to compare high-dimensional classification in order to find dominance species with significant differences among groups. The results demonstrated that Fusobacteria (Fusobacteria to genus) and Lachnospiraceae were the key bacterial types, thus leading to the imbalance of gut microbiota in the HFD group. Lactobacillus, Ruminococcus, and Bacteroidetes (Bacteroidetes to genus) were relatively enriched in three BE treatment groups (Figure 4D). To sum up, these findings indicated that BE can regulate the changes of gut microbiota in HFD-induced rats, and thus exert anti-NAFLD potency.

### Effects of BE on intestinal permeability

**Figure 5:**
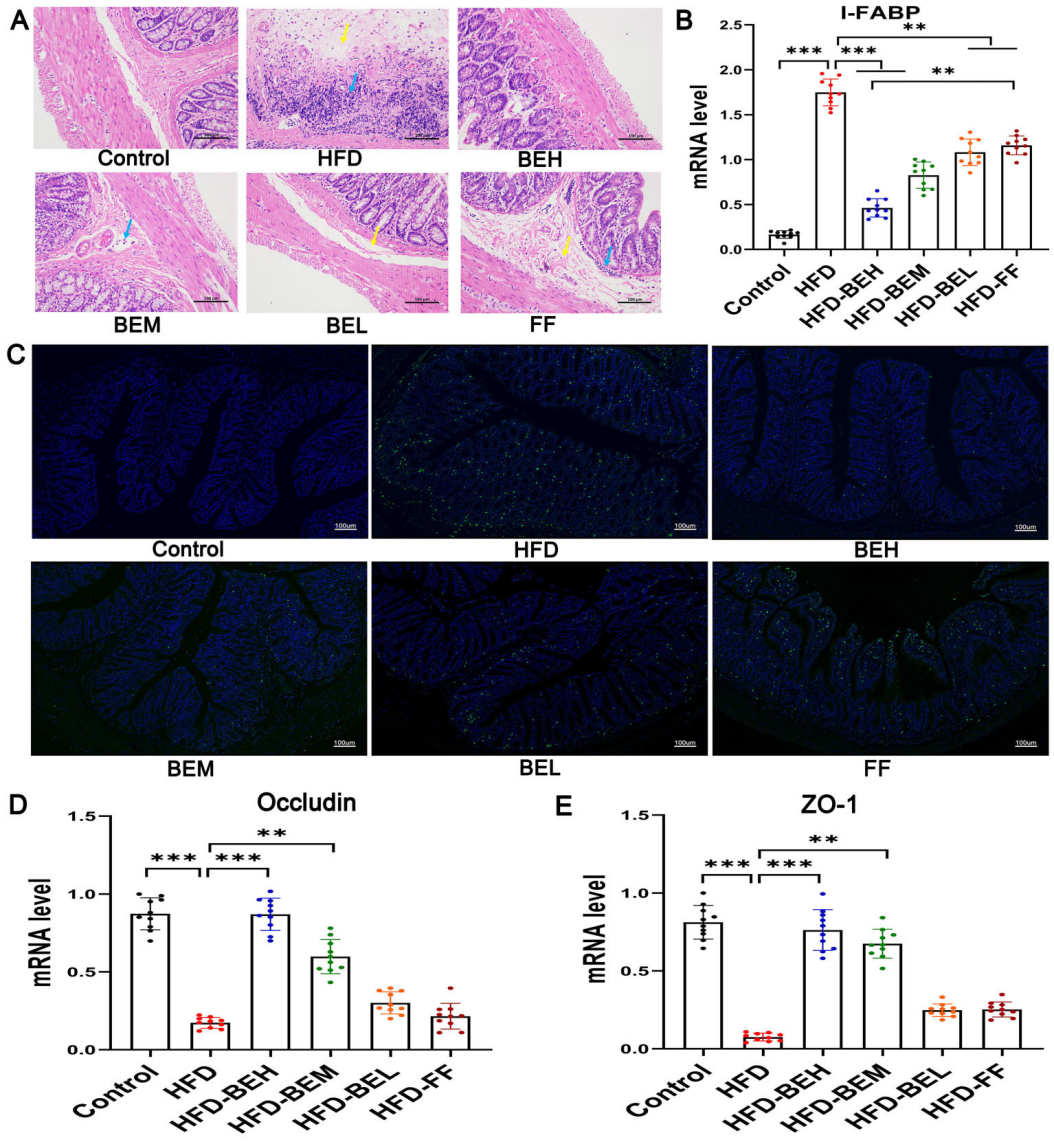
Effect of berberine and evodiamine (BE) at high, medium, and low doses (BEH, BEM, BEL) or fenofibrate (FF) on intestinal integrity of animals fed a high-fat diet (HFD). **A**, Hematoxylin and eosin (HE) staining of ileum tissues (magnification: 200×, scale bar: 100 µm). The blue arrows indicate lymphocyte infiltration, and the yellow arrows indicate submucosa edema. **B**, mRNA expression of intestine fatty acid binding protein (I-FABP) by qRT-PCR. **C**, Immunofluorescence images of terminal deoxynucleotidyl transferase-mediated nick end labeling (TUNEL) (green fluorescence) as an indicator of apoptosis of ileum epithelial cells (magnification: 10×, scale bar: 100 µm). **D**, mRNA expression of occludin and (**E**) zonula occludens-1 (ZO-1) by qRT-PCR. Data are reported as means±SD. **P<0.01; ***P<0.001 (one-way ANOVA).

Firstly, the intestinal tissue pathological results showed that the epithelium of the mucosa layer was intact, and simultaneously, small increased focal lymphocyte infiltration and mild submucosal edema were occasionally observed in a small number of samples in the BE treatment groups (Figure 5A). Distinctively in the HFD group, inflammatory cell infiltration was observed in each layer, and edema and loose connective tissue were observed in the submucosa. Whereas the positive drugs had limited repair effects on pathological injury of intestine, in the FF group, inflammatory cell infiltration was observed in the lamina propria and surrounding lymphatic vessels, and moderate edema and loose connective tissue were observed in local submucosa. These results indicated the limited efficacy of fenofibrate on pathological injury of intestine.

Ileum epithelial cells and the network of tight junction (TJ) proteins were then examined to further verify the integrity of the intestinal mucosal barrier. We detected changes of I-FABP in ileum tissue in HFD-fed rats. The expression of I-FABP in BE treatment groups was observably lower than the HFD group (P<0.001 or P<0.01), and the therapeutic effects of BE at high dosage were better than fenofibrate (P<0.01) (Figure 5B). In addition, we tested the apoptosis of ileum epithelial cells by TUNEL staining. Our results showed that TUNEL-positive apoptotic cells were clearly observed in the HFD group in contrast to the Control group. The treatment with BE significantly decreased TUNEL-positive cells in ileum tissues compared with the HFD and FF groups (Figure 5C), suggesting BE may alleviate HFD-induced apoptosis of ileum epithelial cells, which suggested that BE significantly alleviated HFD-induced apoptosis of ileum epithelial cells. Finally, the experiments on qRT-PCR displayed that BE supplement can clearly upregulate the expression of occludin and zonula occludens-1 (ZO-1), and it was close to the control group and obviously departed from the HFD group, particularly in high (all P<0.001) and medium dosages (P<0.01), which proved the effectiveness of BE on TJ proteins occludin and ZO-1 (Figure 5D, E). In short, these results revealed that BE supplement had a positive influence on the recovery of intestinal integrity and the improvement of intestinal barrier function in HFD-fed rats.

### Effects of BE on inflammation and oxidative stress

**Figure 6:**
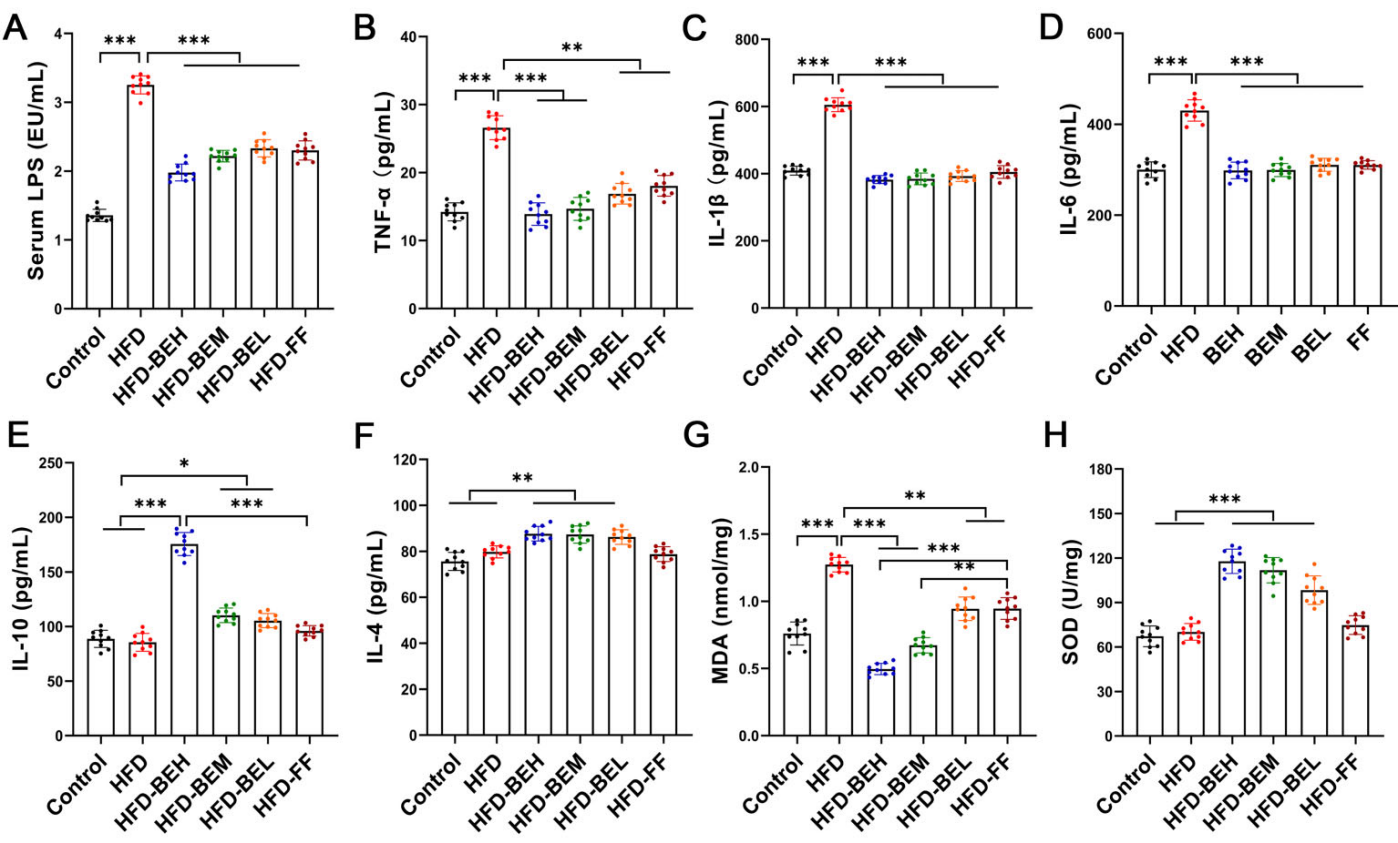
Berberine and evodiamine (BE) at high, medium, and low doses (BEH, BEM, BEL) or fenofibrate (FF) treatment alters inflammatory cytokine expressions and inhibits oxidative stress of animals fed a high-fat diet (HFD). **A**, Lipopolysaccharide (LPS), (**B**) tumor necrosis factor (TNF-α), (**C**) interleukin (IL) 1β, (**D**) IL-6, (**E**) IL-10, and (**F**) IL-4 cytokines levels, (**G**) malondialdehyde (MDA) content, and (**H**) superoxide dismutase (SOD) activity were evaluated by enzyme-linked immunosorbent assay (ELISA). Data are reported as means±SD. *P<0.05, **P<0.01; ***P<0.001 (one-way ANOVA).

We found that the serum LPS levels significantly increased in the HFD group (P<0.001), and the LPS expression levels of BE treatment groups were lower (all P<0.001) (Figure 6A). LPS results in liver inflammation and lipid deposition. We observed that the levels of TNF-α, IL-1β, and IL-6 expression were also high in the HFD group, while the levels in the BE treatment groups were lower and close to the control group (P<0.001 or P<0.01) (Figure 6B-D). In addition, compared with the control and HFD groups, BE treatment significantly increased the levels of the anti-inflammatory cytokines IL-10 (P<0.001 or P<0.05) and IL-4 (all P<0.01), which were significantly better than fenofibrate, especially IL-10 at the high dosage (P<0.001) (Figure 6E, F).

Subsequently, we detected the corresponding representative indicators. The results revealed that the MDA content was significantly elevated in the HFD group (P<0.001); however, BE suppressed this trend and showed better effects than fenofibrate, as high and medium dosages were particularly effective (P<0.001 or P<0.01) (Figure 6G). In addition, BE significantly stimulated SOD activity, which was much higher than that in the control and HFD groups (all P<0.001) (Figure 6H). In brief, the anti-NAFLD effects of BE treatment were related to the inhibition of liver inflammation and oxidative stress.

## Discussion

In recent years, research on NAFLD was mainly concentrated on the theory of enterohepatic axis, in which the interaction between intestinal permeability and gut microbiota are involved in the occurrence and development of NAFLD. Generally, a synergistic effect is obtained when different medicinal plants are combined in traditional Chinese medicine (TCM). However, due to the complexity of comprehensive TCM, its use in modern therapy is limited. Therefore, we explored the mechanisms of the berberine and evodiamine compounds in the cure of HFD-induced NAFLD, and speculated that the effects may be mediated by gut microbiota. The experimental results showed that BE treatment could markedly inhibit weight gain and liver lipid accumulation in HFD-fed rats, and simultaneously attenuate the increase of liver biochemical indexes and pathological changes, which suggested that BE had positive effects on HFD-induced hepatic steatosis and liver injury.

Evidence shows that the alteration of structure and composition of the gut microbia are closely related to NAFLD (1,17). The alpha diversity analysis revealed that BE enhanced the richness and diversity of gut microbiota, yet, the high-dosage group had a smaller effect than the medium-dosage and low-dosage groups identified by previous reports (18). The explanation may be that both berberine and evodiamine are broad-spectrum antibacterial agents that act against many good microorganisms, which was supported by the fact that serum LPS and proinflammatory cytokines were significantly reduced in the high-dosage group (19).

Firmicutes and Bacteroidetes are considered to be the two dominant bacteria in gut microbiota, and the F/B ratio can be used as an indicator of disease, and is related to the munity of the body to disease (20). The data analysis showed that the F/B ratio of the HFD group was significantly higher than that of the control group, which was reversed by BE administration, in line with the conclusions of previous studies (21). The abundance of key genera, involving short-chain fatty acids (SCFAs)-producing Bacteroidetes, Prevotella, and Ruminococcus, was positively correlated with the health status of the gut microbiota (1). The Bacteriodes genus helps sustain the energy metabolism of colon epithelial cells, generates a large number of protective cytokines to maintain the integrity and self-repair of intestinal epithelial cells, and protect the function of the intestinal mucosal barrier (22). Notably, both Prevotella and Ruminococcus are typical intestinal protectors. Lower accumulation of Prevotella in NAFLD patients may lead to increased surface vulnerability and impaired intestinal barrier function (23). Rotman and Sanyal (24) found that from lower Ruminococcus levels to metabolic disease development, circulating acetic acid levels are particularly associated with the Ruminococcaceae-UCG-005 (25). Additionally, Lactobacillus is a kind of probiotics in the intestinal tract that can inhibit reproduction and block the adhesion of pathogenic bacteria and has the function of maintaining the balance of gut microbiota (26).

Lactobacillus has also been shown to lower serum TC and TG (27). In the current study, the relative abundance of Bacteriodes, Ruminococcaceae-UCG-005, Ruminococcaceae-UCG-014, Prevotella-9, and Lactobacillus were increased by BE intervention, which showed that BE prevented the deterioration of NAFLD due in part to the increase of these key bacteria. Specifically, Fusobacteria is a pro-inflammatory gene that causes an imbalance in gut microbiota (28). Proliferation and pathogenic invasion of Fusobacteria have been identified as instrumental factors in Kwashiorkor, while fatty liver has been identified as a specific feature of Kwashiorkor (29). The addition of BE significantly prevented Fusobacteria enrichment in NAFLD rats. Surprisingly, the enrichment of Lachnospiraceae in our study was not consistent with previous reports. Several reports showed that the Lachnospiraceae family is more abundant in healthy subjects than in obese and non-alcoholic steatohepatitis subjects and can even reduce inflammation levels and increase intestinal motility (30). Yet, the conclusions reported by Shen et al. (23) are consistent with our results. They considered Lachnospiraceae as a characteristic fecal bacterium associated with pathogenesis of non-alcoholic steatohepatitis and liver fibrosis. Hence, the specific role of either increased or decreased Lachnospiraceae in HFD-induced NAFLD is uncertain. Collectively, the results indicated that BE could attenuate the deterioration of HFD-induced NAFLD and intestinal injury by selectively increasing certain beneficial bacteria and reducing pathogenic bacteria.

The intestinal mucosa is an important barrier that prevents the intestinal bacteria and their metabolites from entering the vascular system and inducing NAFLD. Moreover, intestinal epithelial cells (IECs) and TJ proteins are important components of the intestinal mucosal barrier. I-FABP is a plasmoprotein expressed in the mucous villous epithelial cells of the ileum, which has been studied as an ideal biomarker for the diagnosis of intestinal injury (31). Occludin, a TJ protein highly concentrated in IECs, is involved in intercellular adhesion, movement, and cell permeability. The presence of occludin determines the gut selective barrier, and occludin levels can reflect damage in the intestinal barrier caused by pathogenic bacteria to a certain extent (32,33). In addition, ZO-1 protein is connected with most TJ proteins and the cytoskeleton, which not only participates in signal transduction of barrier function and maintenance of epithelial cell polarity, but also participates in immune regulation (33). It is worth noting that our many studies on intestinal permeability, including the apoptosis of ileum epithelial cells, the expression of I-FABP and TJ proteins (occludin and ZO-1), showed that BE treatment can reduce intestinal permeability and significantly enhance the intestinal mechanical barrier function.

As gut microbiota is the main source of LPS, the increased intestinal permeability could be the main cause of the significantly elevated LPS levels in portal venous blood and in HFD-induced NAFLD animal models (34). High levels of LPS that enter the blood through the damaged intestinal barrier stimulate the toll-like receptor 4-myeloid differentiation factor 88-nuclear factor kappa B (TLR4-MyD88-NF-ƘB) signaling pathway to induce the activation of downstream related proteins and release pro-inflammatory cytokines (35). TNF-α is the main mediator of the activation of the cascade effect of pro-inflammatory cytokines, and it induces the production of IL-1β, IL-6, and other “secondary” cytokines (36). Il-1 β is associated with the formation of liver fat and liver degeneration, and IL-6 is associated with liver inflammation and fibrosis. However, anti-inflammatory cytokines, such as IL-10 and IL-4, can inhibit the production of pro-inflammatory cytokines, such as TNFα, IL-1β, IL-6, etc. (37). Our studies found that BE intervention can improve the balance between anti-inflammation and pro-inflammation cytokines.

When NAFLD occurs, excessive FFAs participate in β-oxidation to produce reactive oxygen species, including free radicals and hydrogen peroxide, leading to oxidative stress through activated inflammatory pathways (38). MDA, a free radical, can damage the cell membrane by inducing the immune response of liver cells, thus resulting in their structural destruction and dysfunction, and even death. MDA can also activate nuclear transcription factor (NF-KB) to regulate the expression of inflammatory factors and expand the inflammatory response (39). SOD is one of the important enzymes in the biological antioxidant system. It can specifically remove the superoxide free radicals generated in the biological oxidation process and indirectly reflect the antioxidant capacity of tissues (40). The supplementation with BE reduced MDA contents and increased SOD activity. These results suggested that combination therapy with berberine and evodiamine had therapeutic effects for NAFLD by reducing inflammatory overresponse and oxidative stress.

In conclusion, BE modulated gut microbiota, mitigated intestinal permeability, and relieved hepatic inflammatory reaction and hepatic steatosis through the enterohepatic axis as its mechanism to prevent the pathogenesis and evolution of HFD-induced NAFLD. Our findings raised the possibility of using plant alkaloids to control the gut microbiota in the treatment of NAFLD. The potential mechanism of the effects of BE on gut microbiota needs to be further studied, as the role of a number of important gut bacteria have not been well understood and explored, specifically the correlation between pivotal bacteria and other primary indicators of NAFLD.
